# Analyses of the effects of persistent subretinal fluid on visual/anatomic outcomes according to the type of macular neovascularization during the relaxed treat-and-extend protocol in age-related macular degeneration patients

**DOI:** 10.1186/s12886-021-02063-6

**Published:** 2021-08-10

**Authors:** Kyung Tae Kim, Ju Byung Chae, Seungheon Lee, Eoi Jong Seo, Dong Yoon Kim

**Affiliations:** Department of Ophthalmology, Chungbuk National University Hospital, College of Medicine, Chungbuk National University, 776, Sunhwan-1-Ro, Seowon-Gu, Cheongju, 28644 South Korea

**Keywords:** Anti-vascular endothelial growth factor, Macular neovascularization, Exudative age-related macular degeneration, Subretinal fluid

## Abstract

**Background:**

To analyze the long-term effects of persistent subretinal fluid (SRF) on visual/anatomic outcomes according to the type of macular neovascularization (MNV) during relaxed treat-and-extend regimen with anti-vascular endothelial growth factor (anti-VEGF) agents in age-related macular degeneration (AMD) patients.

**Methods:**

Patients with fovea-involving type 1 or type 2 MNV, treated with a relaxed treat-and-extend regimen for 2 years were retrospectively reviewed. Eyes with SRF observed more than three times per year were defined as the ‘persistent SRF (+) group’. To exclude the effects of IRF as much as possible, the eyes with persistent IRF were excluded. The effects of persistent SRF on the best-corrected visual acuity (BCVA), central subfield retinal thickness (CST), and changes in the photoreceptor layer (PRL) thickness and outer retinal bands (external limiting membrane, ellipsoid zone, and cone outer segment tip line) after anti-VEGF injection were analyzed for each MNV type.

**Results:**

Seventy-seven eyes with type 1 MNV (44 eyes with persistent SRF) and 53 eyes with type 2 MNV (18 eyes with persistent SRF) were enrolled. Following a relaxed treat-and-extend regimen with anti-VEGF agents, BCVA and CST improved for each MNV type. In comparison between persistent SRF (+) and persistent SRF (−) group, there were no differences in the amount of change in BCVA and CST between the two groups for each MNV type during 2-year follow-up periods. In addition, there were no differences in the amount of reduction in PRL thickness and state of the outer retinal bands between the two groups for each MNV type.

**Conclusions:**

Using a relaxed treat-and-extend regimen with anti-VEGF agents, persistent SRF did not have additional effects on visual and anatomic outcomes by 2 years, regardless of the MNV type.

## Summary

We analyzed whether persistent SRF affects to visual/anatomic outcomes of exudative AMD according to the MNV type during relaxed treat-and-extend regimen with anti-VEGF agents. We found that small amount of persistent SRF can be tolerated without compromising visual and anatomical outcomes for 2 years, regardless of MNV type.

## Introduction

Optical coherence tomography (OCT) has become a valuable noninvasive retinal imaging modality that provides useful parameters for diagnosis and follow-up monitoring in treatment of patients with exudative age-related macular degeneration (AMD) [1]. Among the various parameters of OCT, the presence of subretinal fluid (SRF) is widely used as a marker of active neovascularization in AMD [2, 3]. Therefore, achieving a completely dry retina was considered as a goal for the treatment of AMD. In addition, the presence of SRF on OCT was used as one of several criteria for retreatment in several large-scale randomized clinical trials [2, 4–6]. Even in clinical practice, treatment decisions for treat-and-extend or pro re nata (PRN) regimen are often driven by the presence of fluid on OCT to reduce the number of anti-vascular endothelial growth factor (anti-VEGF) injections [7].

However, several studies have suggested that resolving SRF completely is not always associated with better visual prognosis [6, 8]. In the Comparison of Age-Related Macular Degeneration Treatments Trials (CATT) study, the greater proportion of patients who achieved a dry retina was not correlated with the proportion of patients who gained visual acuity improvement of more than 15 letters [8]. Another randomized clinical trial reported that there was a minimal difference between the proportions of patients who had visual acuity improvement of more than 15 letters despite the difference in the proportions of patients achieving dry retina [6]. These results question whether it is always necessary to resolve SRF completely in retina when treating AMD.

Several studies have even suggested that SRF may be associated with a better visual prognosis. One study reported that patients with SRF had better visual acuity benefits from anti-VEGF treatment [9, 10], and another study reported that eyes with SRF are less likely to develop retinal pigment epithelium (RPE) atrophy even under intensive anti-VEGF treatment [11]. In addition, a recent study reported that patients treated with a treat-and-extend regimen who tolerated some SRF achieved good visual acuity that is comparable with that achieved when treatment aimed to resolve all SRF completely [12].

Regarding macular neovascularization (MNV) type and its association with visual prognosis, several studies have reported that minimally classic and classic lesions were associated with poorer visual outcomes, and they required more injections of anti-VEGF than occult lesions [13, 14]. These results suggest that the visual prognosis of exudative AMD after anti-VEGF treatment may vary with MNV types. In addition, in our previous study on fibrovascular pigment epithelium detachment (PED) presenting with MNV, we reported that there were no significant differences in the visual and anatomic outcomes, regardless of the presence of persistent SRF, in AMD with type 1 MNV using the relaxed treat-and-extend regimen with anti-VEGF agents for 2 years [15]. From the results of these studies, we have questioned whether the effect of persistent SRF on the visual and anatomic outcomes of AMD is different depending on the MNV type. Therefore, in this study, we aimed to investigate the long-term effect of persistent SRF on visual and anatomic outcomes of AMD patients according to MNV types during a relaxed treat-and-extend regimen with anti-VEGF agents.

## Methods

The records of patients who received an intravitreal anti-VEGF injection for exudative AMD at Chungbuk National University Hospital in Korea between January 2016 and September 2018 were analyzed retrospectively. Approval from the institutional review board and ethics committees of Chungbuk National University Hospital (No. 2020–12-018) was obtained before the initiation of the study, which was performed in compliance with the tenets of the Declaration of Helsinki. We did not obtain patient consent, since data were analyzed anonymously. The institutional review board and ethics committees of Chungbuk National University Hospital (No. 2020–12-018) waived the need for informed consent.

### Inclusion and exclusion criteria

Treatment naïve AMD patients with fovea-involving type 1 or 2 MNV who were treated with a relaxed treat-and-extend regimen with anti-VEGF agents and followed-up for at least 2 years were included in this study. MNV was diagnosed and classified based on fluorescein angiography (FA), indocyanine green angiography (ICGA) and spectral-domain OCT (SD-OCT). We classified MNVs into type 1 and type 2 according to published disease definitions [16]. MNVs containing both type 1 and type 2 lesions were classified as type 2 [17]. The eyes with polypoidal choroidal vasculopathy (PCV) was included in the type 1 MNV group. Patients received three consecutive monthly intravitreal anti-VEGF injections followed by a relaxed treat-and-extend regimen, allowing treatment extension by 2 weeks depending on disease activity (up to a maximum extension interval of 12 weeks) [18]. The disease activity during anti-VEGF treatment was defined as follow (1) a loss of BCVA of 5 letters of more than the best BCVA recorded since baseline, (2) new retinal hemorrhage, (3) presence of fluid on SD-OCT, or (4) a combination of the aforementioned. Fluid was defined as the presence of any intraretinal fluid (IRF) (resulting from disease activity as judged by the retinal specialists) or any SRF of more than 200 μm in height at the foveal center. Subfoveal SRF with a height of 200 μm or less or any SRF elsewhere was tolerated, and it did not prohibit extension [12]. The exclusion criteria included type 3 MNV, geographic atrophy (GA) or fibrovascular scar at the macula, and any history of photodynamic therapy or macular laser therapy. To exclude the effects of IRF as much as possible, the eyes with persistent IRF were excluded. Other ocular conditions that could compromise visual acuity or affect image quality were also excluded.

### Definition of ‘persistent SRF’

SRF observed more than three times per year was defined as ‘persistent SRF’ [3]. ‘Persistent IRF’ was also defined in the same way. The patients were divided into two groups according to the presence of persistent SRF for each MNV type. Eyes with persistent SRF during anti-VEGF therapy were allocated to the ‘persistent SRF (+) group’ and the others were allocated to the ‘persistent SRF (-) group’. The two groups were compared to investigate whether persistent SRF affects visual or anatomic outcomes for each MNV type.

### Outcome measurement

At the initial visit, all patients underwent a comprehensive ophthalmic examination, including BCVA assessment using the Snellen chart, intraocular pressure (IOP) measurement, slit-lamp examination, color fundus photography, FA, ICGA and SD-OCT (Spectralis; Heidelberg Engineering, Heidelberg, Germany). At each subsequent visit, patients underwent ophthalmic examinations, including the assessment of BCVA, applanation tonometry, slit-lamp examination, dilated fundus examination, fundus photography, and SD-OCT [15].

The BCVA and CST of the persistent SRF (+) and persistent SRF (−) groups before the anti-VEGF injections, at 1 month after initial three loading injections (4 months), 6, 12, 18, and 24 months after the injections were compared for each MNV type. In addition, the number of intravitreal anti-VEGF injections, choroidal thickness, and outer retinal changes such as changes in the photoreceptor layer (PRL), external limiting membrane (ELM), ellipsoid zone (EZ), and cone outer segment tip (COST) line in the two groups after the injections for 2 years were compared for each MNV type [15].

### SD-OCT analysis

**Fig. 1:**
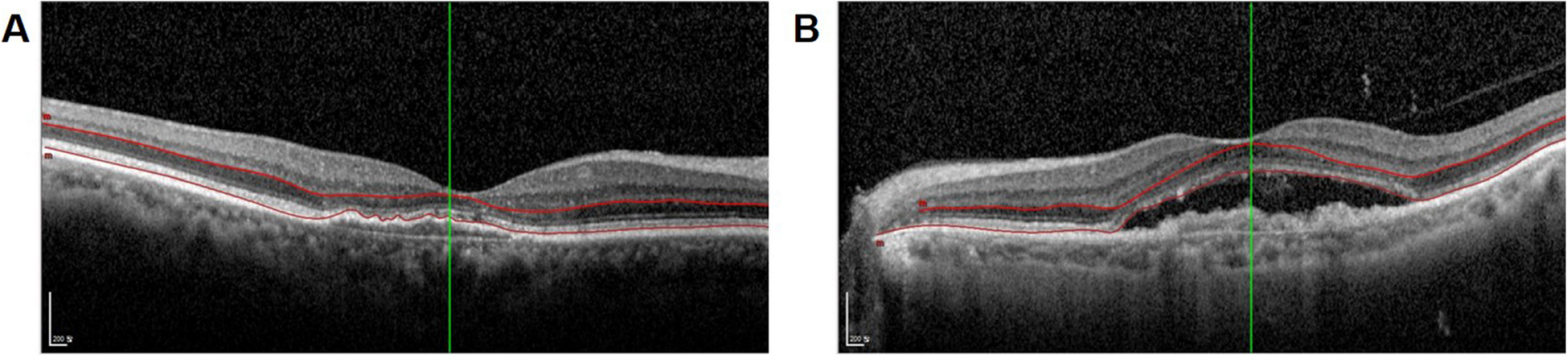
Segmentation the retinal layer and measuring the photoreceptor layer (PRL) thickness. Segmenting the anterior margin of the retinal pigment epithelium (RPE) and the outer margin of the outer plexiform layer (OPL) was manually performed using the segmentation software. The PRL thickness was measured automatically after the segmentation through the software built into SD-OCT. The PRL thickness was measured as the distance from the outer margin of the OPL to the anterior margin of the RPE in eyes without SRF (**A**). In eyes with SRF, the PRL thickness was measured as the distance from the outer margin of the OPL to the outer end of the photoreceptors (**B**)

To analyze the anatomic outcomes, the central subfield thickness (CST), subfoveal choroidal thickness (SFCT), PRL thickness, and outer retina bands (ELM, EZ, and COST line) were investigated by SD-OCT. The protocol for images of SD-OCT followed the previously introduced method, which is as follows [19]. The volumetric scans of Spectralis SD-OCT were acquired with Spectralis Viewing Module (Version 6.0.9.0). For images obtained by Spectralis SD-OCT, a custom 20° × 20° volume acquisition protocol which covered 6 mm × 6 mm surface of the macula was used to obtain one set of high-speed scans form each eye. With this protocol, 49 cross-sectional B-scan images were obtained, each composed of 512 A-scans. The integrated follow-up mode of the device was used to ensure that the exact same retinal area was imaged at every follow-up visit [19]. The PRL thickness was measured as the distance from the outer margin of the outer plexiform layer (OPL) to the anterior margin of the RPE [20]. If there was SRF at the fovea, the PRL thickness was measured as the distance from the outer margin of the OPL to the outer end of the photoreceptors [21]. Segmenting the outer margin of the OPL and the anterior margin of the RPE on SD-OCT image was manually performed using the segmentation software built into SD-OCT by two different retinal specialists (JBC and EJS) who were masked to the study design. The PRL thickness was measured automatically after the segmentation through the built-in software. The values of PRL thickness at 3 locations, such as fovea center and 500 μm away from fovea center, were averaged (Fig. 1). The average of both measurements from each retinal specialist was used for the analysis.

### Statistical analysis

Data are presented as the mean ± SD or numbers (%). The Kolmogorov-Smirnov test was used for assessing normality. The statistical analyses were conducted using SPSS version 22.0 software (SPSS, Inc., Chicago, IL, USA). A *P*-value < 0.05 was considered statistically significant.

The paired *t*-test was used to evaluate the differences in BCVA and CST between the baseline and last visit. And the Student’s t-test was used to evaluate the differences in parameters including BCVA, CST, PRL thickness and the number of injections between the ‘persistent SRF (+) group’ and ‘persistent SRF (-) group’ for each CNV type. The differences in the status of the ELM, EZ, and COST line between groups was analyzed by Pearson’s χ^2^ test.

## Results

A total of 77 eyes from 77 patients with type 1 MNV and 53 eyes from 53 patients with type 2 MNV were included in this study (12 eyes from type 2 CNV group were excluded because persistent IRF was observed). Among 77 eyes with type 1 MNV, 28 eyes with PCV were included. The demographics and baseline ocular findings of the patients are summarized in Table 1. The mean ages of patients were 74.2 ± 7.2 years for type 1 MNV group and 76.6 ± 7.3 years for type 2 MNV group. The mean baseline BCVA of type 1 MNV group was better than that of type 2 MNV group (0.54 ± 0.30 in type 1 MNV group, 0.75 ± 0.36 in type 2 MNV group, *p* = 0.001), and the mean baseline SFCT of type 2 MNV group was lower than that of type 1 MNV group (293.64 ± 79.97 in type 1 MNV group, 229.32 ± 76.76 in type 2 MNV group, *p* < 0.001). Bevacizumab in 13 eyes, ranibizumab in 17 eyes, and aflibercept in 47 eyes were used for the initial loading treatment in the type 1 MNV group. In the type 2 MNV group, bevacizumab in 5 eyes, ranibizumab in 20 eyes, and aflibercept in 28 eyes were used for the initial loading treatment. In each group, 16 eyes were treated with two or more types of anti-VEGF after the initial loading treatment. The prevalence of SRF or IRF at baseline significantly differed in the two groups (77 eyes with SRF in type 1 MNV, 49 eyes with SRF in type 2 MNV, *p* = 0.014; 2 eyes with IRF in type 1 MNV, 39 eyes with IRF in type 2 MNV, *p* < 0.001). There were no significant differences in the mean baseline CST and the number of intravitreal anti-VEGF injections between the two groups.

**Table 1:** Patient demographics and baseline ocular findings in type1 CNV and type 2 CNV group

Characteristics.	Type 1 CNV group (*n* = 77)	Type 2 CNV group (*n* = 53)	*p*-value
Age, years (mean ± SD)	74.2 ± 7.2	76.6 ± 7.3	0.067
Sex, male/female (%)	51/26 (66/34)	29/24 (55/45)	0.072
Lens status, phakic/pseudophakic (%)	57/20 (74/26)	33/20 (62/38)	0.153
Best-corrected visual acuity, logMAR (mean ± SD)	0.54 ± 0.30	0.75 ± 0.36	0.001
Central subfield retinal thickness, μm (mean ± SD)	398.06 ± 123.07	436.74 ± 131.05	0.089
Subfoveal choroidal thickness, μm (mean ± SD)	293.64 ± 79.97	229.32 ± 76.76	< 0.001
Anti-VEGF, n (%)			0.324
Bevacizumab	10 (13)	5 (9)	
Ranibizumab	12 (15)	12 (23)	
Aflibercept	39 (51)	20 (38)	
Mixed	16 (21)	16 (30)	
No. of intravitreal anti-VEGF injections (mean ± SD)			
1 year	7.0 ± 1.6	6.8 ± 1.4	0.436
2 year	5.1 ± 1.9	5.1 ± 1.4	0.940
Total	12.0 ± 3.0	11.8 ± 2.4	0.631
Fluid on OCT at baseline, n (%)			
SRF	77 (100)	49 (92)	0.014
IRF	2 (3)	39 (74)	< 0.001

*CNV* choroidal neovascularization, *SD* standard deviation, *logMAR* logarithm of the minimal angle of resolution, *VEGF* vascular endothelial growth factor, *OCT* optical coherence tomography

### Comparison of the persistent SRF (+) and persistent SRF (−) groups for each MNV type

We divided the eyes into two groups according to the presence of persistent SRF in type 1 MNV and type 2 MNV groups. There were 44 eyes in the persistent SRF (+) group and 33 eyes in the persistent SRF (−) group in the type 1 MNV group. The number of intravitreal anti-VEGF injections during the follow-up did not differ in the two groups in the type 1 MNV group. In addition, there were no significant differences in the BCVA, CST, SFCT, the state of outer retinal bands and the mean change of PRL thickness between the two groups for 2 years (Table 2). In type 1 MNV group, analysis of PED status was performed. The heights of PED in the persistent SRF (+) and persistent SRF (−) group were 175.80 ± 104.82 and 196.25 ± 105.81, respectively (*p* = 0.403). In the persistent SRF (−) group, the complete regression of PED was observed in 3 eyes, and there were no eyes with complete regression of PED in the persistent SRF (+) group. The amounts of changes of PED height in the persistent SRF (+) and persistent SRF (−) group were − 32.62 ± 71.15 and − 87.66 ± 93.68, respectively (*p* = 0.007).

**Table 2:** Visual/anatomic outcomes between ‘persistent SRF (+) group’ and ‘persistent SRF (-) group’ in type 1 CNV and type 2 CNV

Characteristics.	Type 1 CNV		*p*	Type 2 CNV		*p*
	Persistent SRF (+) group (*n* = 44)	Persistent SRF (−) group (*n* = 33)		Persistent SRF (+) group (*n* = 18)	Persistent SRF (−) group (*n* = 35)	
No. of intravitreal anti-VEGF injections (mean ± SD)						
1 year	7.2 ± 1.5	6.7 ± 1.7	0.175	7.3 ± 1.4	6.5 ± 1.4	0.059
2 year	5.4 ± 1.7	4.7 ± 2.0	0.092	5.4 ± 1.8	4.9 ± 1.1	0.276
Total	12.6 ± 2.7	11.3 ± 3.3	0.073	12.7 ± 2.7	11.4 ± 2.1	0.061
Values at baseline (mean ± SD)						
BCVA, logMAR	0.52 ± 0.27	0.58 ± 0.35	0.453	0.70 ± 0.30	0.78 ± 0.39	0.443
CST, μm	386.95 ± 119.15	412.88 ± 128.44	0.364	446.11 ± 121.83	431.91 ± 137.02	0.713
SFCT, μm	305.03 ± 78.74	276.78 ± 72.40	0.142	264.31 ± 76.03	211.26 ± 71.79	0.023
Values at 2 years after anti-VEGF injections (mean ± SD)						
BCVA, logMAR	0.35 ± 0.25	0.35 ± 0.26	0.900	0.42 ± 0.28	0.55 ± 0.35	0.192
CST, μm	303.02 ± 57.34	275.45 ± 67.67	0.057	314.28 ± 67.56	269.11 ± 53.84	0.011
SFCT, μm	294.14 ± 77.97	262.64 ± 73.66	0.077	249.94 ± 77.36	200.46 ± 75.45	0.029
ELM intact/defect	41/3	29/4	0.423	14/4	19/16	0.095
EZ intact/defect	23/21	19/14	0.644	8/10	8/27	0.105
COST line intact/defect	1/43	2/31	0.395	1/17	0/35	0.159
Δ PRL thickness from After 3rd injection	−5.70 ± 2.66	−6.58 ± 2.45	0.145	−6.22 ± 3.72	−8.40 ± 3.84	0.054

*CNV* choroidal neovascularization, *SRF* subretinal fluid, *VEGF* vascular endothelial growth factor, *SD* standard deviation, *BCVA* best-corrected visual acuity, *logMAR* logarithm of the minimal angle of resolution, *CST* central subfield thickness, *SFCT* subfoveal choroidal thickness, *ELM* external limiting membrane, *EZ* ellipsoid zone, *COST* con outer segment tip, *PRL* photoreceptor layer

In type 2 MNV group, 18 eyes were classified into the persistent SRF (+) group whereas 35 eyes were classified into the persistent SRF (−) group. The number of intravitreal anti-VEGF injections administered during the follow-up did not differ in the two groups. The mean SFCT in the persistent SRF (+) group was significantly higher than that in the persistent SRF (−) group at baseline and after 2 years. In addition, the mean CST in the persistent SRF (+) group was higher than that in the persistent SRF (−) group. However, there were no significant differences in the BCVA, the state of outer retinal bands, and the mean change of PRL thickness between the two groups for 2 years (Table 2).

### Changes in BCVA and CST

**Fig. 2:**
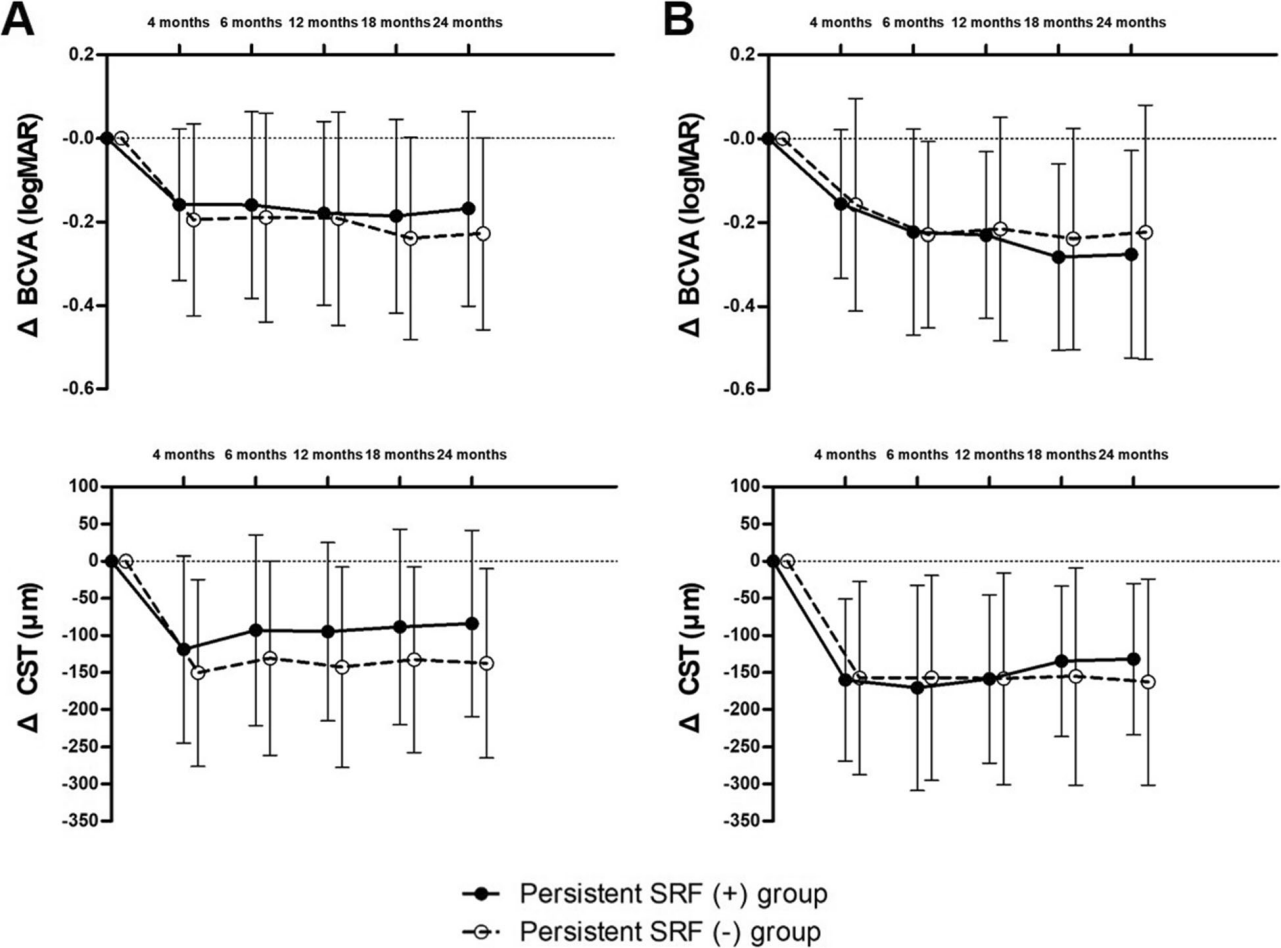
Graph illustrating changes in the amount of decrease in best-corrected visual acuity (BCVA) and central subfield retinal thickness (CST). Both type 1 MNV (**A**) and type 2 MNV (**B**) groups show significant improvement in the BCVA and CST after 2 years, regardless of the presence of persistent SRF. The BCVA and CST changes from baseline with the relaxed treat-and-extend regimen did not differ in the persistent SRF (+) and persistent SRF (−) groups for both type 1 and type 2 MNV during the 2-year follow-up

With the treat-and-extend treatment strategy, the BCVA and CST were significantly improved and maintained in both persistent SRF (+) and persistent SRF (−) groups for each MNV type. For the type 1 MNV group, the BCVAs of the persistent SRF (+) and persistent SRF (−) groups were improved to 0.35 ± 0.25 (*p* < 0.001) and 0.35 ± 0.26 (*p* < 0.001), respectively, from the baseline after 2 years. The CST was improved to 303.02 ± 57.34 in the persistent SRF (+) group (*p* < 0.001) and 275.45 ± 67.67 in the persistent SRF (−) group (*p* < 0.001) after 2 years. For the type 2 MNV group, the BCVAs of the persistent SRF (+) and persistent SRF (−) groups were improved to 0.42 ± 0.28 (*p* < 0.001) and 0.55 ± 0.35 (*p* < 0.001) from baseline after 2 years, respectively. The CST after 2 years was improved to 314.28 ± 67.56 in the persistent SRF (+) group (*p* < 0.001) and 269.11 ± 53.84 in the persistent SRF (−) group (*p* < 0.001). The BCVA and CST changes from baseline was presented in Fig. 2. The amount of BCVA and CST changes during the-2-year follow-up did not differ between the persistent SRF (+) and persistent SRF (−) groups in the type 1 groups (BCVA, *p* = 0.756; CST, *p* = 0.085). In the type 2 MNV group, the amount of BCVA and CST changes during the-2-year follow-up did not differ between the persistent SRF (+) and persistent SRF (−) groups (BCVA, *p* = 0.532; CST, *p* = 0.407).

### Changes in PRL thickness

**Fig. 3:**
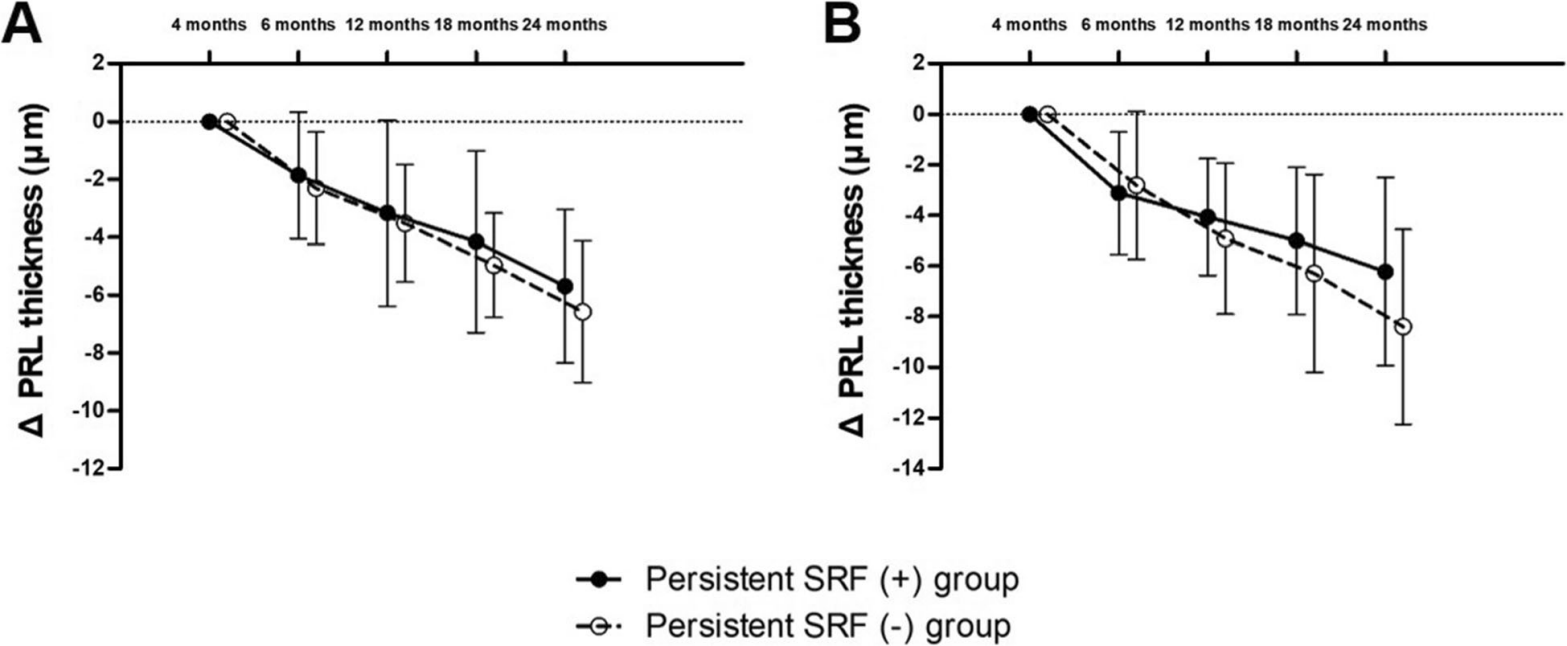
Graph illustrating changes in the amount of decrease in the photoreceptor layer (PRL) thickness. Both type 1 MNV (**A**) and type 2 MNV (**B**) groups show a continuous reduction in PRL thickness, and the amount of decrease in PRL thickness did not differ in the persistent SRF (+) and persistent SRF (−) groups for both type 1 and type 2 MNV during the 2-year follow-up

**Fig. 4:**
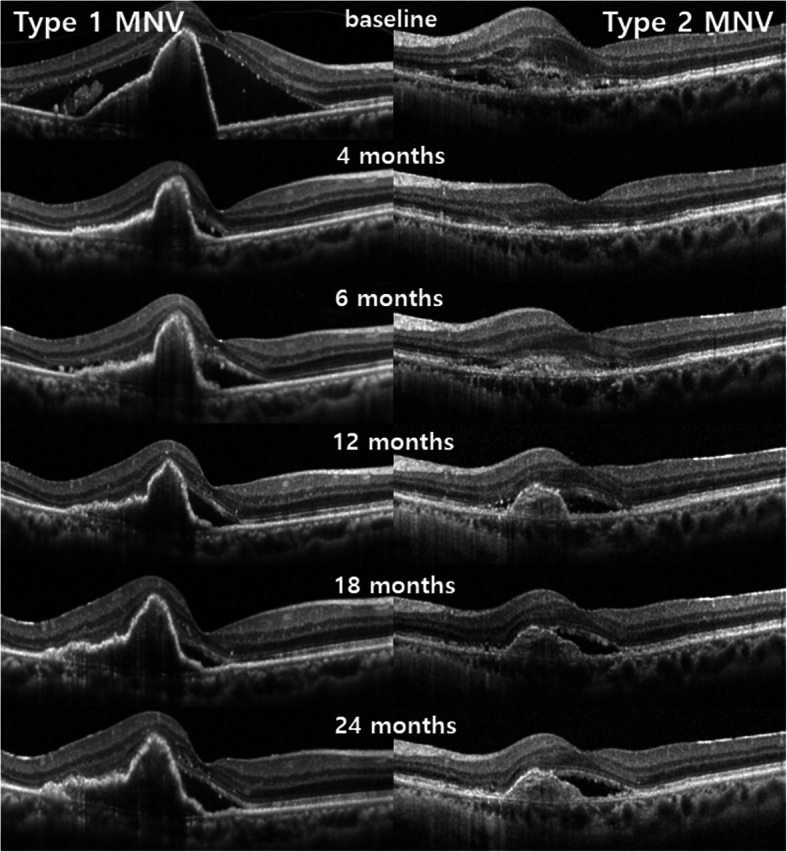
Optical coherence tomography (OCT) images of the representative cases with persistent SRF from the type 1 and type 2 MNV groups. The first case (left column) is of a 74-year-old male with type 1 MNV treated using the treat-and-extend regimen with aflibercept during the 2-year follow-up. The Snellen BCVA was 0.3, and fibrovascular PED and SRF were observed on SD-OCT at baseline. A total 13 times of intravitreal aflibercept injection were administered for 2 years. Although small amount of SRF persisted for 2 years, the Snellen BCVA was improved to 0.5 and apparent atrophic or degenerative change in the outer retina were not observed for 2 years. The second case (right column) is of a 68-year-old female with type 2 MNV treated using the treat-and-extend regimen with aflibercept during the 2-year follow-up. The Snellen BCVA was 0.5, and subretinal MNV, SRF and IRF were observed on SD-OCT at baseline. A total 12 times of intravitreal aflibercept injection were administered for 2 years. The SRF and IRF were subsided after 3-monthly aflibercept intravitreal injections, but SRF recurred at 6 months and persisted. Although persistent SRF was observed, the Snellen BCVA was improved to 0.7 and external limiting membrane and ellipsoid zone were intact at 2 years

During the follow-up, the PRL thickness continuously decreased in the persistent SRF (+) and persistent SRF (−) groups for each MNV type (Fig. 3). For type 1 MNV, the amount of decrease in PRL thickness during the-2-year follow-up was not different between the persistent SRF (+) and persistent SRF (−) groups (*p* = 0.145). For type 2 MNV, the slope of the decrease in PRL thickness in the persistent SRF (−) group tended to be steeper than that of the persistent SRF (+) group. However, the amount of decrease in PRL thickness was not different between the two groups during the 2-year follow-up (*p* = 0.054). Representative cases with persistent SRF from type 1 and type 2 MNV groups are shown in Fig. 4.

### Sub-analysis of eyes with PCV in type 1 MNV group

Among 77 eyes with type 1 MNV, 28 eyes with PCV were included (17 eyes in the persistent SRF (+) group and 11 eyes in the persistent SRF (−) group). The total number of intravitreal anti-VEGF injections administered during the follow-up was 12.8 ± 1.6 in the persistent SRF (+) group (7.0 ± 1.1 at 1 year, 5.8 ± 1.2 at 2 year). In the persistent SRF (−) group, the total number of intravitreal anti-VEGF injections was 12.0 ± 3.1 (7.5 ± 1.9 at 1 year, 4.6 ± 2.0 at 2 year). There were no significant differences in the number of intravitreal anti-VEGF injections between the two groups (*p* = 0.452 at 1 year, *p* = 0.059 at 2 years, *p* = 0.428 for 2 years).

With the treat-and-extend treatment strategy, the BCVA and CST were significantly improved and maintained in both persistent SRF (+) and persistent SRF (−) groups. The BCVAs of the persistent SRF (+) and persistent SRF (−) group were improved 0.44 ± 0.22 (*p* = 0.034) and 0.33 ± 0.30 (*p* = 0.008), respectively, from the baseline after 2 years. The CST was improved to 326.82 ± 56.53 in the persistent SRF (+) group (*p* = 0.002) and 273.45 ± 75.68 in the persistent SRF (−) group (*p* = 0.003) after 2 years. The amount of BCVA and CST changes during the-2-year follow-up did not differ between the persistent SRF (+) and persistent SRF (−) groups in the eyes with PCV (BCVA, *p* = 0.251; CST, *p* = 0.861).

During the follow-up, the PRL thickness continuously decreased in the persistent SRF (+) and persistent SRF (−) groups. The amounts of decrease in PRL thickness during the-2-year follow-up were − 6.41 ± 1.46 and − 6.64 ± 2.69 in the persistent SRF (+) and persistent SRF (−) groups, respectively. There was no significant difference in the amount of decrease in PRL thickness during the 2-year follow-up (*p* = 0.804).

## Discussion

In this study, we investigated the effects of persistent SRF on the visual and anatomic outcomes of eyes with exudative AMD according to the MNV type. The primary finding was that BCVA and CST were improved and well maintained for 2 years in both type 1 and type 2 MNV groups using the relaxed treat-and-extend regimen with anti-VEGF agents, regardless of the presence of persistent SRF. The second finding was that the number of intravitreal anti-VEGF injections in the persistent SRF (+) and persistent SRF (−) groups did not differ with the relaxed treat-and-extend regimen. Furthermore, anatomical changes in the outer retina, including changes in the outer retinal bans and PRL thickness, were not different in both MNV groups, regardless of the presence of persistent SRF.

The presence of fluid on OCT is used widely as a marker of activity of the neovascular process [2, 3]. In line with this, several studies have reported the negative impact of SRF on visual outcome. Hoerster et al. reported that SRF correlated significantly with impaired BCVA [22], and Golbaz et al. reported that SRF was more frequently associated with recurrent disease [23]. In addition, one study demonstrated that retinal alteration like SRF affect negatively to retinal sensitivity by using microperimetry [24]. On the other hand, the evidence that SRF may be associated with a positive effect on VA has recently been supported by several studies. Some studies have reported that the presence of SRF at any time is associated with higher VA levels [9, 25]. Other studies have demonstrated that patients with SRF have better visual acuity benefits from anti-VEGF therapy [9, 10]. In addition, Sato et al. reported that eyes with SRF are less likely to develop RPE atrophy [11]. As a possible reason for the positive effects of SRF on visual prognosis, one study suggested that SRF could be suggestive of a functional perfused neovascular net providing RPE and PRL survival in contrast to advanced vascular atrophy in the sub-RPE space [26]. In this study, we investigated the effect of persistent SRF on visual and anatomic outcomes in eyes with exudative AMD. The final BCVA and the degree of improvement in BCVA in the persistent SRF (+) group were not different significantly from those in the persistent SRF (−) group. In addition, the persistent SRF had no additional effects on the outer retina with the relaxed treat-and-extend regimen during the 2-year follow-up period. In other words, the persistent SRF (+) group, compared with the persistent SRF (−) group, showed non-inferior visual and anatomic outcomes for 2 years with the relaxed treat-and-extend regimen. These results suggest that persistent SRF can be tolerated without compromising visual and anatomic outcomes during exudative AMD treatment with the relaxed treat-and-extend regimen by 2 years, regardless of the MNV type. Figure 4 shows representative cases with persistent SRF from the type 1 and type 2 MNV groups.

Unlike SRF, the negative effects of IRF on BCVA were demonstrated consistently by several studies [25, 27, 28]. Ritter et al. reported that eyes with IRF presented with the lowest initial VA, and IRF had the strongest negative predictive value for functional improvement [27]. And other studies demonstrated that eyes with IRF show reduced initial visual acuity by a mean of two lines on ETDRS charts [9, 25, 28]. Unfortunately, we could not investigate the effect of persistent IRF, because the main purpose of this study was to investigate the effect of persistent SRF for each MNV type. Therefore, we excluded the eyes with persistent IRF to minimize the effect of IRF on visual and anatomic outcomes. In addition, the eyes with persistent IRF were relatively rare because IRF caused by exudative MNV has been known that they are exquisitely responsive to anti-VEGF treatment [26]. Bolz et al. also reported an almost complete reduction of exudative IRF 1 week after a single anti-VEGF injection [29]. In current study, IRF was resolved completely after 3-monthly intravitreal anti-VEGF injection in both type 1 and type 2 CNV groups. Persistent IRF was observed in only 12 eyes despite anti-VEGF treatment, and they were excluded from the type 2 MNV group. Considering that RPE atrophy or scarring were observed in all these 12 eyes, the IRF observed in these eyes was considered degenerative IRF and not related to exudative MNV but neurosensory degeneration [26].

In multiple randomized trials, eyes treated with fixed-dosing anti-VEGF injection had better visual acuity at 1 and 2 years than those that received less frequent injections [2, 30, 31]. However, a monthly treatment regimen may increase the overall treatment burden for patients. Therefore, to minimize the injection frequency, a proactive dosing regimen, such as the treat-and-extend regimen, was introduced [32]. Several studies reported that the treat-and-extend regimen was statistically noninferior and clinically comparable with a monthly regimen in improving VA for all three types of anti-VEGF [33–35]. Furthermore, recent randomized clinical trial used extension criteria to extend the injection interval even when a small amount of SRF was observed with the treat-and-extend regimen [12]. This study demonstrated that patients treated with a ranibizumab treat-and-extend regimen who tolerated some SRF (subfoveal SRF of 200 μm or less) achieved VA comparable with that achieved when treatment aimed to resolve all SRF completely [12]. In current study, a relaxed treat-and-extend regimen, which allows subfoveal SRF of 200 μm or less, was used as treatment regimen, and we confirmed that the BCVA and CST were improved and well maintained for 2 years in both type 1 and type 2 MNV groups, regardless of the persistent SRF. In addition, considering that there was no difference in the number of anti-VEGF injections between the persistent SRF (+) and persistent SRF (−) groups for each MNV type, it was expected that a relaxed treat-and-extend regimen could reduce the number of anti-VEGF injections even in the presence of tolerable SRF. However, the exact amount and nature of tolerable SRF have not been established for the treat-and-extend regimen for the treatment of exudative AMD. Therefore, a prospective study is warranted to investigate the exact extent and properties of tolerable SRF for anti-VEGF treatment of exudative AMD.

In this study, the proportion of eyes with persistent SRF was higher for type 1 MNV than for type 2 MNV. This might be explained by the varying fluid patterns with the MNV type. A study reported that SRF is the predominant form of exudation and the first typical exudative sign in type 1 MNV lesions, and the presence of IRF in type 1 MNV lesions presumably indicates damage to the outer blood-retinal barrier in the form of RPE dysfunction and disruption in the tight junctions that contribute to the ELM band on SD-OCT. [16] This study also reported that IRF predominates with type 2 MNV lesions rather than SRF [16]. Similarly, in current study, SRF was observed in all the eyes with type 1 MNV, and IRF was more frequently observed in eyes with type 2 MNV than those with type 1 MNV on the image of initial SD-OCT. In addition, the proportion of eyes with persistent SRF was different between the type 1 (57%) and type 2 MNV (34%) groups.

A strength of the current study is that it is the first study to analyze the effect of persistent SRF on visual and anatomic outcomes according to MNV type. Through this analysis, we confirmed that persistent SRF did not affect the visual or anatomic outcomes additionally during anti-VEGF treatment with the relaxed treat-and-extend regimen in both type 1 and type 2 MNV lesions by 2 years. Therefore, it is considered that a favorable outcome can be achieved with a relatively small number of anti-VEGF injections even in eyes with persistent SRF regardless of MNV type. However, this study has some notable limitations that are inherent in its retrospective and nonrandomized design with a small sample size. Second, the number of anti-VEGF injections received by each patient and the drug type (bevacizumab, ranibizumab, and aflibercept) were not controlled; thus, a heterogeneous population was enrolled. Third, the eyes with PCV were included in the type 1 MNV group, and some mixed case which have both type 1 and type 2 MNV were included in the type 2 MNV group. Fourth, with the exclusion of the eyes with persistent IRF despite of anti-VEGF therapy, patients with relatively mild exudative AMD may have been included in the type 2 MNV group. Fifth, since a quantitative analysis of SRF has not been conducted, the quantitative criteria for tolerable SRF in anti-VEGF treatment have not been provided. Sixth, due to the relatively short-term follow-up period of 2 years, it might be difficult to determine the effects of persistent SRF on visual function, requiring long-term follow-up studies. Finally, the characterization of visual function in the included patients may have been relatively poor because objective tests for visual function were not performed. Using visual function tests such as static perimetry or multifocal ERG may facilitate more objective evaluation of patients’ visual function; thereby, we would better understand the relationship between functional visual acuity and anatomical changes in AMD patients. Therefore, further prospectively designed studies with large sample sizes using objective visual function tests are warranted. In addition, quantitative and qualitative studies to investigate the criteria of tolerable SRF also be needed in the future.

In conclusion, we found that visual and anatomical prognoses were relatively good and well maintained using a relaxed treat-and-extend regimen, regardless of the presence of persistent SRF in both type 1 and type 2 MNV. And these results of this study were obtained by excluding eyes with persistent IRF. Using a relaxed treat-and-extend regimen with anti-VEGF agents, persistent SRF might be tolerated without compromising visual and anatomic outcomes by 2 years, regardless of the MNV type.

## Data Availability

The data used to support the findings of this study are restricted by the Institutional Review Board of Chungbuk National University Hospital in order to protect PATIENT PRIVACY. Data are available from Dong Yoon Kim (umlover9@gmail.com) for researchers who meet the criteria for access to confidential data.

## Abbreviations

SRF: Subretinal fluid
MNV: Macular neovascularization
VEGF: Vascular endothelial growth factor
AMD: Age-related macular degeneration
RPE: Retinal pigment epithelium
BCVA: Best-corrected visual acuity
CST: Central subfield retinal thickness
SFCT: Subfoveal choroidal thickness
ELM: External limiting membrane
EZ: Ellipsoid zone
COST: Cone outer segment tip
PED: Pigment epithelium detachment
OPL: Outer plexiform layer
